# Bicolor angelfish (*Centropyge bicolor*) provides the first chromosome-level genome of the Pomacanthidae family

**DOI:** 10.46471/gigabyte.32

**Published:** 2021-11-09

**Authors:** Chunhua Li, Xianwei Yang, Libin Shao, Rui Zhang, Qun Liu, Mengqi Zhang, Shanshan Liu, Shanshan Pan, Weizhen Xue, Congyan Wang, Chunyan Mao, He Zhang, Guangyi Fan

**Affiliations:** ^1^ BGI-Qingdao, BGI-Shenzhen, Qingdao 26655-5, China; ^2^ Department of Biology, Hong Kong Baptist University, Hong Kong, China; ^3^ College of Life Sciences, University of Chinese Academy of Sciences, Beijing 100049, China

## Abstract

The Bicolor Angelfish, *Centropyge bicolor*, is a tropical coral reef fish. It is named for its striking two-color body. However, a lack of high-quality genomic data means little is known about the genome of this species. Here, we present a chromosome-level *C. bicolor* genome constructed using Hi-C data. The assembled genome is 650 Mbp in size, with a scaffold N50 value of 4.4 Mbp, and a contig N50 value of 114 Kbp. Protein-coding genes numbering 21,774 were annotated. Our analysis will help others to choose the most appropriate *de novo* genome sequencing strategy based on resources and target applications. To the best of our knowledge, this is the first chromosome-level genome for the Pomacanthidae family, which might contribute to further studies exploring coral reef fish evolution, diversity and conservation.

## Data description

### Background

**Figure 1.**
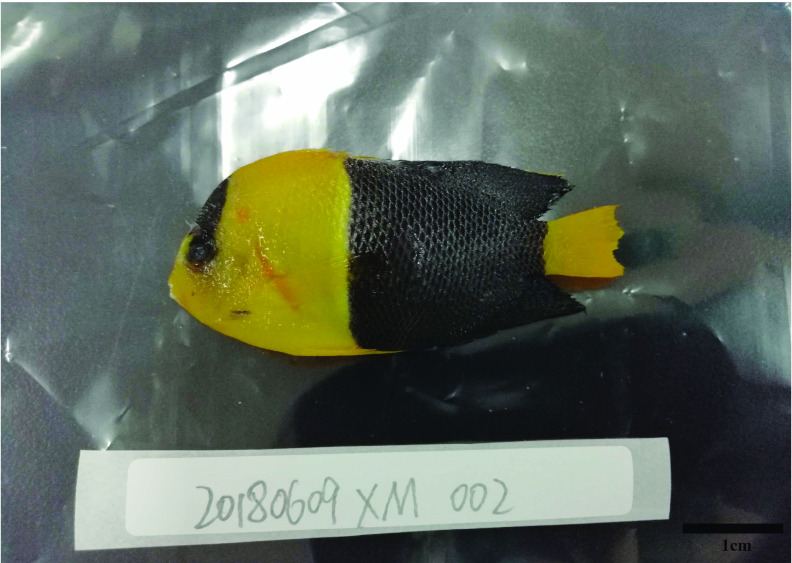
Photograph of *Centropyge bicolor.*

*Centropyge bicolor* (NCBI:txid109723; FishbaseID: 5454; urn:lsid:marinespecies.org:taxname:211780) (Figure 1), also known as the Bicolor, Two-Colored, or Pacific Rock Beauty Angelfish, is a showy coral reef fish commonly distributed in the Indo–Pacific ocean (from East Africa to the Samoan and Phoenix Islands, north to southern Japan, south to New Caledonia; throughout Micronesia). As a member of the Pomacanthidae family, it is similar to those of the Chaetodontidae (Butterflyfishes) but is distinguished by the presence of strong preopercle spines. *C. bicolor* has clear boundaries between its body colors, so might be a good model in which to study body color development in coral fish [1].

### Context

Although the availability of genetic, and especially genomic resources, remains limited for the Pomacanthidae family, we assembled the first *C. bicolor* reference genome. This will provide valuable information for genetic studies of this coral reef fish, and will contribute to studies in body color diversity. With the whole genome sequence of *C. bicolor*, it might be possible to explore the genetic mechanisms of body color development in coral reef fish by comparative genomic methods.

## Methods and results

**Figure 2.**
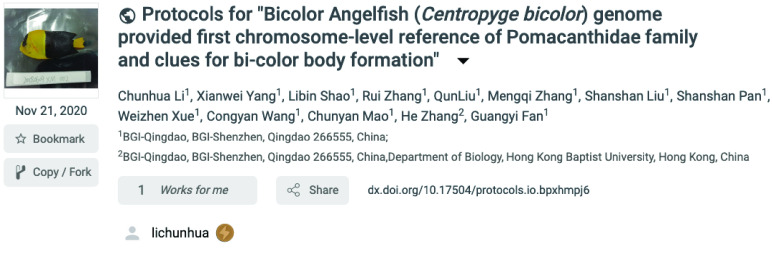
Protocols for BGISEQ-500, stLFR and Hi-C library preparation and construction, and genome assembly, for the Bicolor Angelfish, *Centropyge bicolor* [2]. https://www.protocols.io/widgets/doi?uri=dx.doi.org/10.17504/protocols.io.bpxhmpj6

A protocols collection for BGISEQ-500, stLFR and Hi-C library construction is available in protocols.io (Figure 2) [2].

### Sample collection and genome sequencing

A *C. bicolor* individual was collected from the market in Xiamen, Fujian Province, China. DNA was extracted from fresh muscle tissue according to a standard protocol. Single-tube long fragment read (stLFR) [2] and Hi-C libraries were constructed following the manufacturers’ instructions [2, 3] to sequence and assemble the genome. We obtained 130.47 Gbp (gigabase pairs; ∼197×) raw stLFR data and 134.57 Gbp (∼203.20×) raw Hi-C data (Table 1) using the BGISEQ-500 platform in 100-bp (basepair) paired-end mode.

Low-quality reads (sequences with more than 40% of bases with a quality score lower than 8), polymerase chain reaction (PCR) duplications, adaptor sequences and reads with a high (greater than 10%) proportion of ambiguous bases (Ns) occurring in stLFR data were filtered using SOAPnuke (v1.6.5; RRID:SCR_015025) [4]. We obtained 62.6 Gbp (∼91.67×) clean data (Table 1) to assemble the draft genome. Meanwhile, HiC-Pro (v. 2.8.0) [5] was used for the quality control of raw Hi-C data, and 42.51 Gbp (∼64.19×) valid data were used to assemble the genome to the chromosome-level (Table 1).

**Figure 3.**
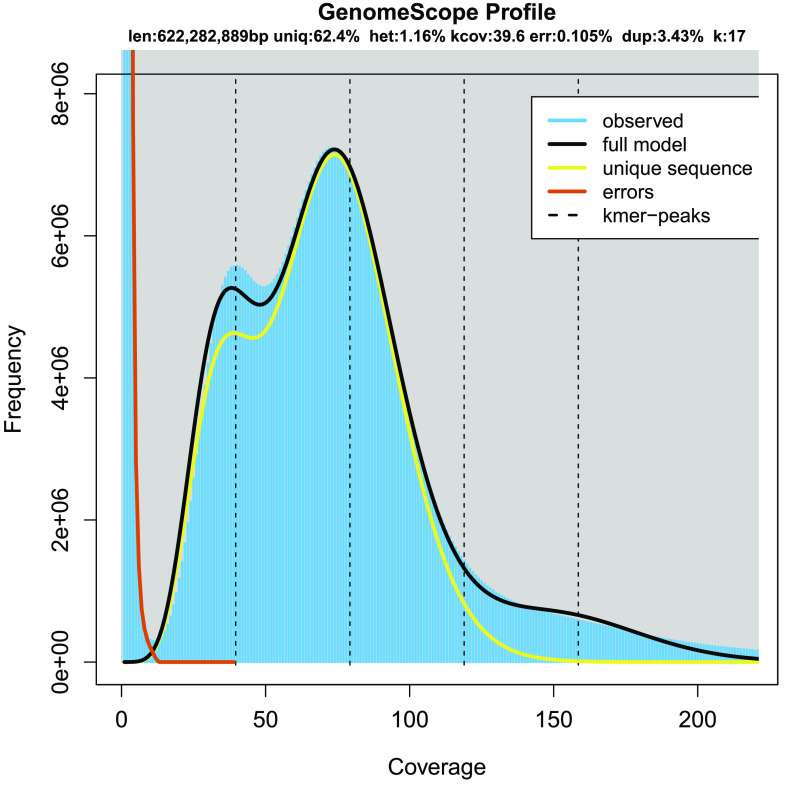
The 17-mer depth distribution of *Centropyge bicolor*. The estimated genome size is 662.27 Mbp and the heterozygosity is 1.16%.

**Table 1:** Statistics of DNA sequencing data.

		Raw data		Valid data	
Libraries	Read length	Total bases (Gbp)	Sequencing depth (×)	Total bases (Gbp)	Sequencing depth (×)
stLFR	100:100	130.47	197.00	60.71	91.67
Hi-C	100:100	134.57	203.20	42.51	64.19

Sequencing depth = Total bases / Genome size, where the genome size is the result of *k*-mer estimation, as shown in Table 2.

### Genome assembly

Using GenomeScope software (RRID:SCR_017014) with stLFR clean data, *k*-mer distribution was used to understand the genome complexity before genome assembly [6]. The genome size of *C. bicolor* was estimated as 662.27 Mbp (megabase pairs), with 37.6% repeat sequences and 1.16% heterozygous sites (Table 2, Figure 3).

**Table 2:** Statistical information of 17-mer analysis.

*k*-mer	*k*-mer number	*k*-mer Depth	Heterozygosity (%)	Genome size (Mbp)
17	50,994,645,240	77	1.16	662.27

The genome size, G, was defined as G = K_num_/K_depth_, where K_num_ is the total number of *k*-mers, and K_depth_ is the most frequently occurring *k*-mer.

We reformatted the clean stLFR data into 10× Genomics format using an in-house script [7] and assembled the draft genome using Supernova (v.2.0.1, RRID:SCR_016756) [8] with default parameters. The draft genome was 681 Mbp, with a contig N50 of 115.5 Kbp (kilobase pairs) and scaffold N50 of 4.4 Mbp (Table 3), which is similar to the estimated genome size.

**Table 3:** Statistics of the draft assembly with stLFR data.

Statistics	Contig	Scaffold
Total number (#)	40,442	29,065
Total length (bp)	655,705,062	681,285,455
Gap (N) (bp)	0	25,580,393
Average length (bp)	16,213.47	23,440.06
N50 length (bp)	115,524	4,424,004
N90 length (bp)	6,029	7,618
Maximum length (bp)	1,148,507	21,943,074
Minimum length (bp)	48	940
GC content (%)	41.74	41.74

**Figure 4.**
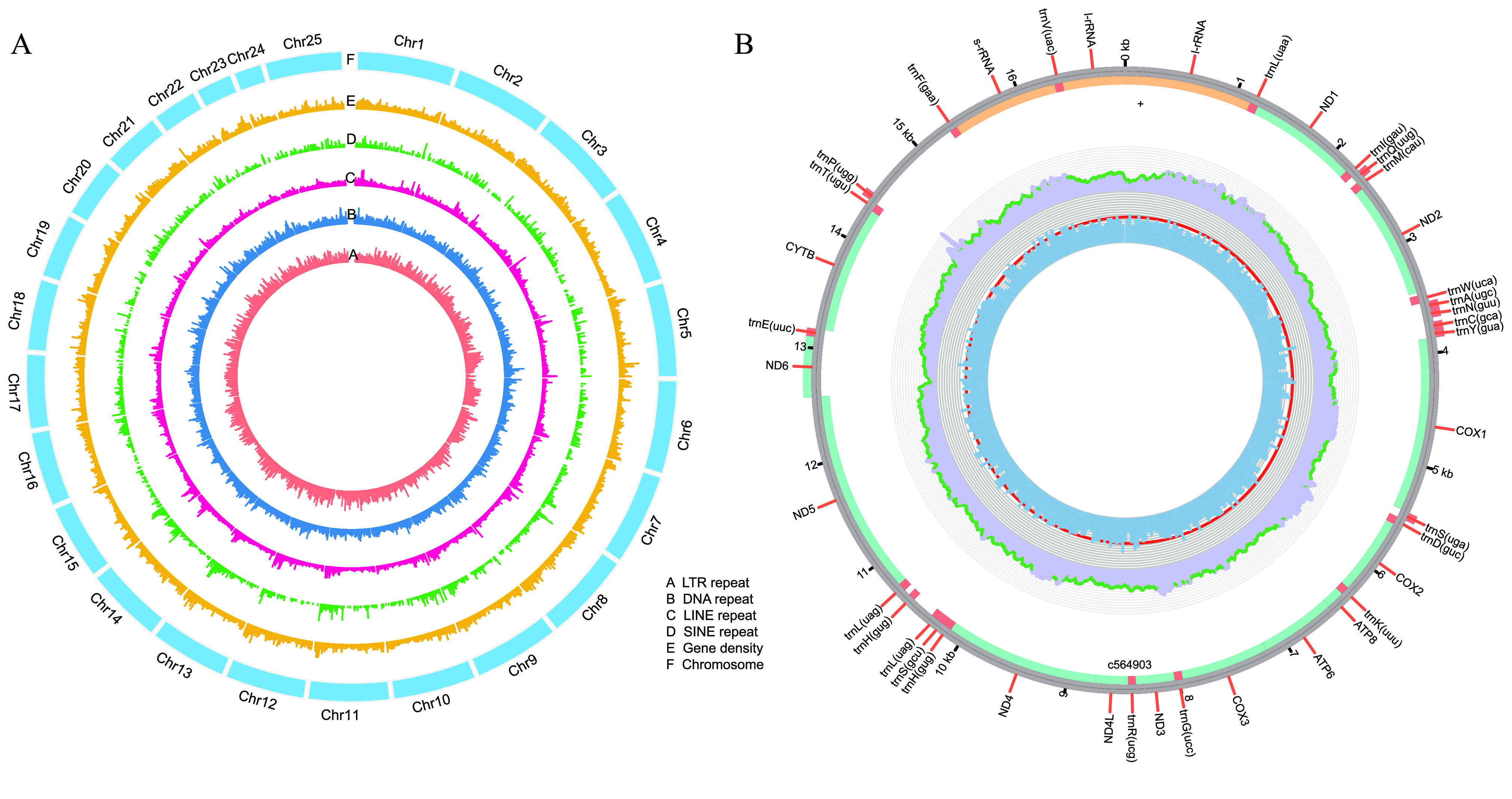
Annotation of the *Centropyge bicolor* genome. (A) Basic genomic elements of the *Centropyge bicolor* genome. LTR, long terminal repeat; LINE, long interspersed nuclear elements; SINE, short interspersed elements. (B) Physical map of mitochondrial assembly.

**Figure 5.**
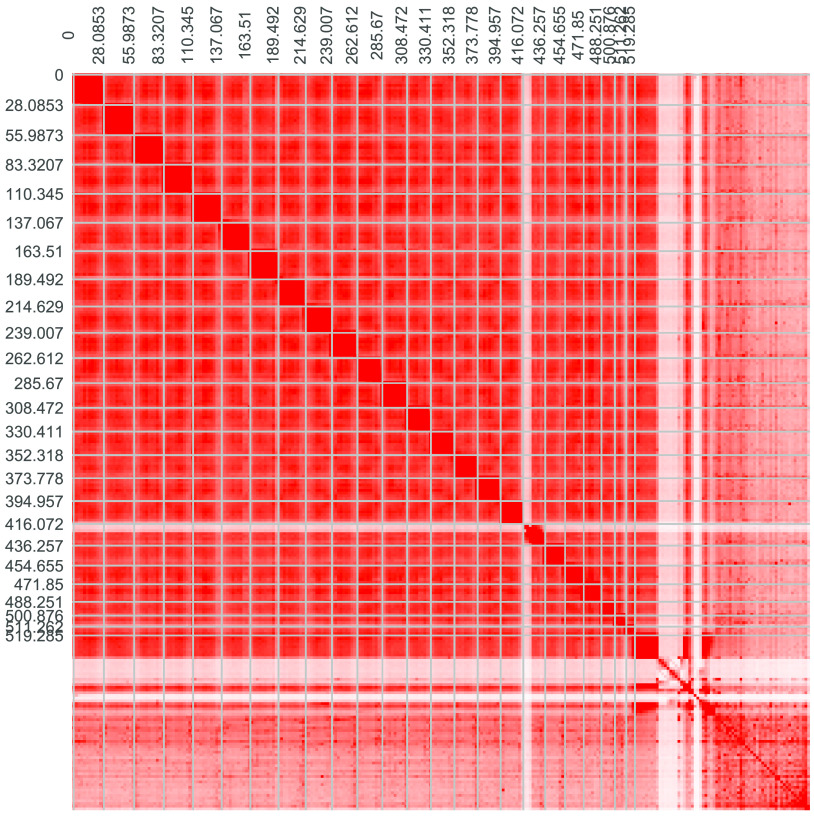
Heat map of interactive intensity between chromosome sequences.

**Figure 6.**
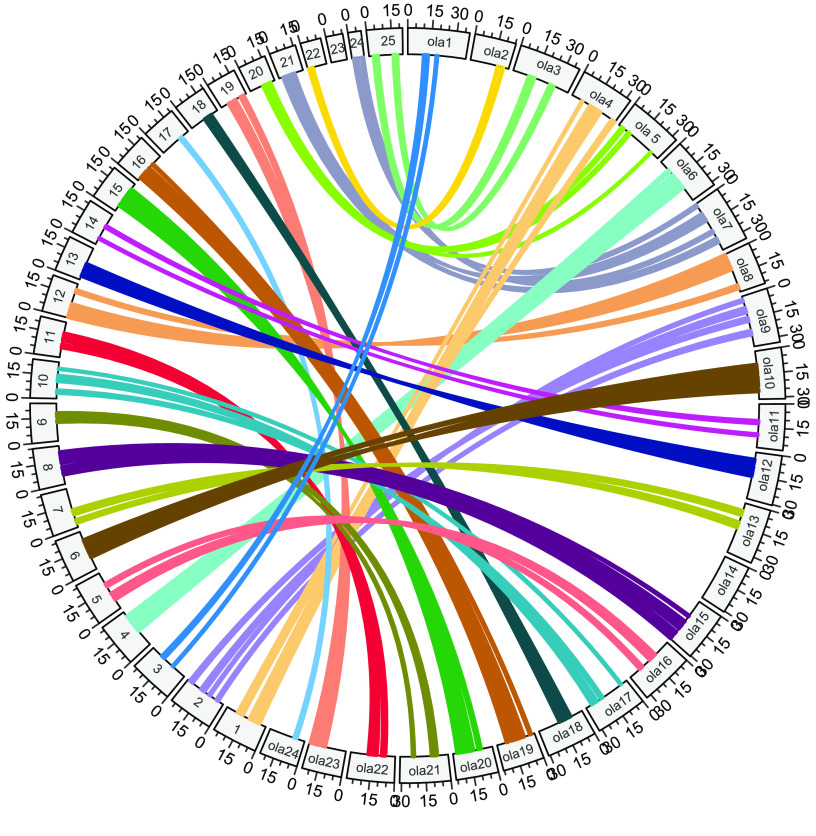
Homologous chromosomal regions between *Centropyge bicolor* and *Oryzias latipes*.

To obtain the chromosome-level genome, we used Juicer (v3, RRID:SCR_017226) [9] to build a contact matrix and 3dDNA (v.170123) [10] to sort and anchor scaffolds with the parameters: “–m haploid –s 4 –c 24”. There are 24 distinct contact blocks, which correspond to 24 chromosomes, representing 96% of the whole genome (Figures 4A, 5, Table 4). On evaluating the completeness of the genome and gene set using Benchmarking Universal Single-Copy Orthologs (BUSCO, v.3.0.2, RRID:SCR_015008) [11] and a vertebrata database, our assembly maintained a score of 96.2% (Table 5). We also identified putative homologous chromosomal regions between *C. bicolor* and *Oryzias latipes* by MCscanx [12] (Figure 6).

In addition, we cut off partial stLFR reads (25 M) for assembly by MitoZ with default parameters [13] and obtained a 16,961-bp circular mitochondrial genome of *C. bicolor*. Thirteen protein-coding genes, 24 tRNA genes and three rRNA genes were annotated by GeSeq (RRID:SCR_017336) [14] (Figure 4B).

### Genomic annotation

For the annotation of repeats, we carried out homolog annotation and *ab initio* prediction independently. RepeatMasker (v.4.0.6, RRID:SCR_012954) [15], RepeatProteinMask (a module from RepeatMasker) and trf (Tandem Repeats Finder, v.4.07b) [16] were used to identify known repetitive sequences by comparing the whole genome with RepBase [17]. LTR_FINDER (v.1.06, RRID:SCR_015247) [16, 18] and RepeatModeler (v.1.0.8, RRID:SCR_015027) [19] were used in *de novo* prediction. We also classified transposable elements (TEs) from the integration of all repeats. In total, we identified 124 Mbp (18.32% of the entire genome) of repetitive sequences (Figure 4A, Table 6), including 110 Mbp of TEs (Figure 4A, Table 7).

**Table 4:** Statistics of the chromosome-level genome.

Statistics	Contig	Scaffold
Total number (#)	40,778	28,555
Total length (bp)	655,705,062	680,873,932
Gap (N) (bp)	0	25,168,870
Average length (bp)	16,079.87	23,844.30
N50 length (bp)	113,563	21,943,074
N90 length (bp)	5,988	7,542
Maximum length (bp)	1,148,507	28,105,280
Minimum length (bp)	43	43
GC content (%)	41.74	41.74

**Table 5:** Statistics of the BUSCO assessment.

Types of BUSCOs	Gene set		Assembly	
	Number	Percentage (%)	Number	Percentage (%)
Complete BUSCOs	2,408	93.1	2,486	96.2
Complete single-copy BUSCOs	2,348	90.8	2,438	94.3
Fragmented BUSCOs	81	3.1	64	2.5
Missing BUSCOs	97	3.8	36	1.3
Total BUSCO groups searched	2,586	100	2,586	100

**Table 6:** Statistics of repetitive sequences.

Type	Repeat size (bp)	Percentage of genome (%)
TRF	14,165,095	2.08
RepeatMasker	43,423,877	6.38
RepeatProteinMask	12,503,750	1.84
*De novo*	110,871,693	16.28
Total	124,708,977	18.32

**Table 7:** Statistics of transposable elements.

	Repbase TEs, *n* (%)	Protein TEs, *n* (%)	*De novo* TEs, *n* (%)	Combined TEs, *n* (%)
DNA	27,163,851 (3.990)	1,068,990 (0.157)	61,731,447 (9.067)	70,925,963 (10.417)
LINE	10,228,332 (1.502)	6,956,340 (1.022)	20,006,579 (2.938)	26,714,285 (3.924)
SINE	856,125 (0.126)	0 (0.000)	497,024 (0.073)	1,187,676 (0.174)
LTR	10,971,817 (1.611)	4,485,808 (0.659)	16,270,071 (2.390)	23,101,529 (3.393)
Other	10,041 (0.001)	0	0	10,041 (0.001)
Unknown	0	0	14,054,230 (2.064)	14,054,230 (2.064)
Total	43,423,877 (6.378)	12,503,750 (1.836)	99,265,690 (14.579)	109,868,166 (16.136)

Homolog-based and *ab initio* prediction were used to identify the protein-coding genes. Augustus (v.3.3, RRID:SCR_008417) [20] was used in *ab initio* prediction basing on a repeat-masked genome [21]. Protein sequences of *Astatotilapia calliptera*, *Danio rerio*, *Larimichthys crocea*, and *Oreochromis niloticus* were downloaded from the National Center for Biotechnology Information (NCBI) GenBank database and aligned to the *C. bicolor* genome for homolog gene annotation with Genewise (v2.4.1, RRID:SCR_015054) [22]. Finally, we used GLEAN [23] to integrate all the above evidence and obtained a total of 21,774 genes, which contained 11 exons on average and had an average coding sequence (CDS) length of 1,575 bp (Table 8).

**Figure 7.**
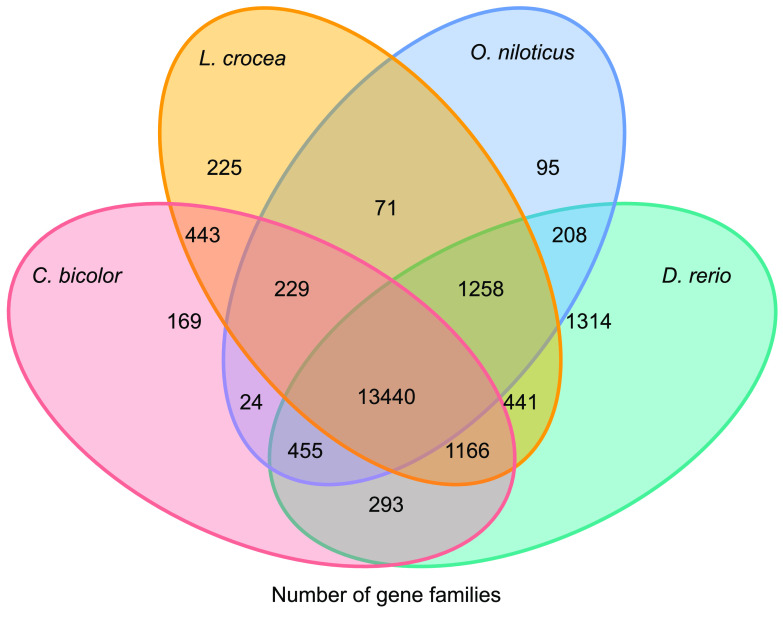
Venn diagram of orthologous gene families. Four teleost species (*Centropyge. bicolor, Larimichthys crocea, Oreochromis niloticus, and Danio rerio*) were used to generate the Venn diagram based on gene family cluster analysis.

To predict gene functions, 21,774 genes were aligned against several public databases, including TrEMBL [24], SwissProt [24], KEGGViewer [25] and InterProScan [26]. As a result, 99.67% of all genes were predicted functionally (Table 9, Figure 7).

**Table 8:** Statistics of the predicted genes in the bicolor angelfish genome.

	Gene set	Gene number	Average transcript length (bp)	Average CDS length (bp)	Average intron length (bp)	Average exon length (bp)	Average exons per gene
Homolog	*Astatotilapia calliptera*	51,174	21,762.29	2,259.23	1,691.33	180.29	12.53
	*Danio rerio*	22,005	27,982.75	1,570.36	3,438.82	180.90	8.68
	*Larimichthys crocea*	47,419	19,884.78	2,139.39	1,575.94	174.50	12.26
	*Oreochromis niloticus*	47,067	17,771.04	1,906.97	1,608.29	175.53	10.86
*De novo*	Augustus	34,470	9,675.42	1,335.20	1,344.81	185.40	7.20
GLEAN		21,774	14,024.40	1,906.28	1,206.07	172.55	11.05

The GLEAN gene set is the integrated result of *de novo* gene predictions and homolog gene predictions.

### Phylogenetic analysis

We downloaded the gene data of seven representative teleost fishes from NCBI to study the phylogenetic relationships between *C. bicolor*. These seven fishes were: *Danio rerio*, *Gasterosteus aculeatus*, *Gadus morhua*, *Larimichthys crocea*, *Oryzias latipes*, *Oreochromis niloticus* and *Tetraodon nigroviridis*. For each dataset, the longest transcripts were selected and aligned to each other by BLASTP (v2.9.0, RRID:SCR_001010) [27] (*E*-value ≤ 1e-5). TreeFam (v.2.0.9, RRID:SCR _013401) [28] was used to cluster gene families, with default parameters. Among all 20,706 clustered gene families, there were 4,450 common single-copy families and 57 families specific to *C. bicolor* (Table 10). With single-copy sequences, we used PhyML (v.3.3, RRID:SCR_014629) [29] to construct the phylogenetic tree of *C. bicolor* and the seven other fishes mentioned above, setting *D. rerio* as an outgroup.

**Figure 8.**
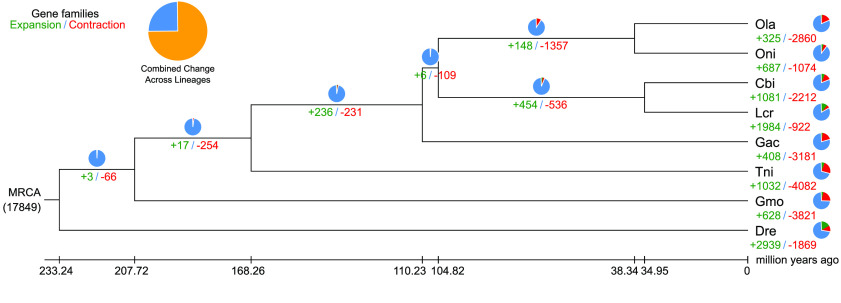
Comparative analysis of the *Centropyge bicolor* genome. (A) The protein-coding genes of the eight species were clustered into 17,849 gene families. Among these gene families, 4,450 were single-copy gene families. (B) Phylogenetic analysis of *Centropyge bicolor* (Cbi.), *Danio rerio* (Dre.), *Gasterosteus aculeatus* (Gac.), *Gadus morhua* (Gmo.), *Larimichthys crocea* (Lcr.), *Oryzias latipes* (Ola.), *Oreochromis niloticus* (Oni.), and *Tetraodon nigroviridis* (Tni.) using single-copy gene families. The species differentiation time between *Centropyge bicolor* and *Larimichthys crocea* was ∼34.95 million years.

**Table 9:** Statistics of the functional annotation.

Database	Number	Percentage (%)
Total	21,774	100.00
SwissProt	20,784	95.45
KEGG	19,168	88.03
TrEMBL	21,688	99.61
Interpro	20,153	92.56
Overall	21,702	99.67

**Table 10:** Statistics of gene family clustering.

Species	Total genes	Unclustered genes	Families	Unique families	Average number of genes per family
*Centropyge bicolor*	21,774	694	16,219	57	1.3
*Danio rerio*	30,067	2,188	18,575	726	1.5
*Gasterosteus aculeatus*	20,756	784	15,921	16	1.25
*Gadus morhua*	19,987	535	15,630	9	1.24
*Larimichthys crocea*	24,403	610	17,273	55	1.38
*Oryzias latipes*	19,535	1,048	14,805	87	1.25
*Oreochromis niloticus*	21,431	180	15,780	14	1.35
*Tetraodon nigroviridis*	19,544	901	14,803	57	1.26

Based on the phylogenetic tree and single-copy sequences, the divergence time between different species was estimated by MCMCTREE with parameters of “–model 0 –rootage 500 -clock 3”. The results showed that *C. bicolor* was formed ∼34.95 million years ago, when differentiated from the common ancestor with *L. crocea* (Figure 8).

### Analysis of bicolor formation in teleosts

**Figure 9.**
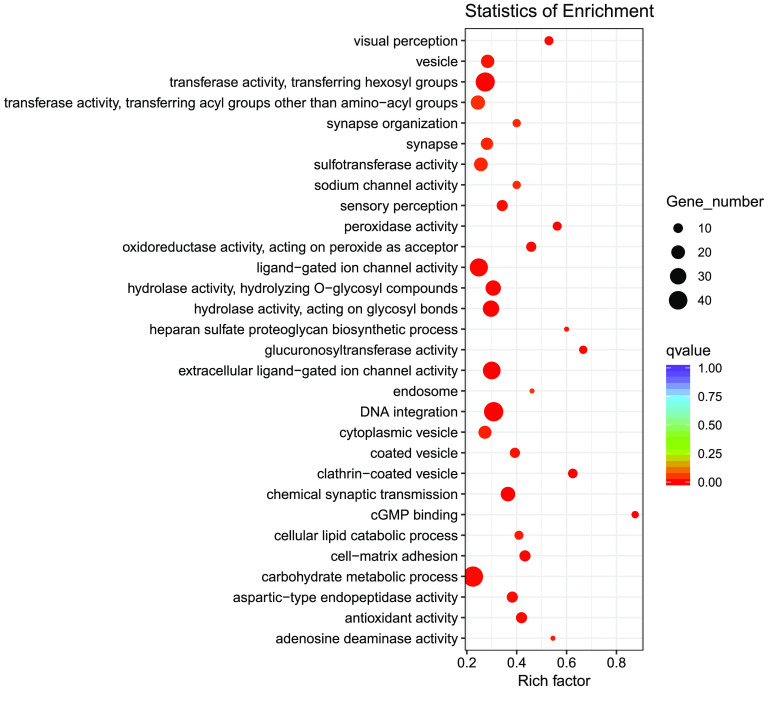
Statistics of gene function enrichment (Gene Ontology) for expanded genes of *Centropyge bicolor*. Nodes are colored by *q*-value (adjusted *p*-value). Node size is shown according to its enriched gene number.

Current studies suggest that different pigment cells produce different pigments. Some types of pigment cells already have been identified in teleost [30]. *C. bicolor* has an attractive body color with clear color boundaries, but the molecular mechanism underlying this remains unknown. Compared with other teleost, there are 1,081 expanded gene families and 57 specific gene families in *C. bicolor* (Figure 9). Functional enrichment analysis showed that notable expansion occurred in those gene families related to visual development and enzyme metabolism (Figure 9).

## Re-use potential

Coral reef fishes, with distinctive color patterns and color morphs, are important for understanding the adaptive evolution of fishes. In this study, we firstly assembled a high-quality, chromosome-level genome of *C. bicolor*, with a length of 681 Mbp, and annotated 21,774 genes. This is the first genome of a fish from the Pomacanthidae family. These genomic data will be useful for genome-scale comparisons and further studies on the mechanisms underlying colorful body development and adaptation.

## Data Availability

The data sets supporting the results of this article are available in the *GigaScience* Database [31]. Raw reads from genome sequencing and assembly are deposited at the China National Gene Bank under reference number CNP0001160, which contains sample information (CNS0315939), Hi-C raw data (CNX0286336) and stLFR raw data (CNX0286337). The project also has been deposited at NCBI under accession ID PRJNA702283.

## List of abbreviations

bp: base pair; BUSCO: Benchmarking Universal Single-Copy Orthologs; Gbp: gigabase pair; Kbp: kilobase pair; KEGG: Kyoto Enyclopedia of Genes and Genomes; Mbp: megabase pair; NCBI: National Center for Biotechnology Information; stLFR: single-tube long fragment reads; TE: transposable element.

## References

[1] Mendoncą RC, Chen JY, Zeng C, Tsuzuki MY, Embryonic and early larval development of two marine angelfish, *Centropyge bicolor* and *Centropyge bispinosa*. Zygote, 2020; 28(3): 196–202. doi:10.1017/S0967199419000789. 32083523

[2] Li C Protocols for “Bicolor Angelfish (*Centropyge bicolor*) genome provided first chromosome-level reference of Pomacanthidae family and clues for bi-color body formation”. protocols.io. 2020; 10.17504/protocols.io.bpxhmpj6.

[3] Wang O Efficient and unique cobarcoding of second-generation sequencing reads from long DNA molecules enabling cost-effective and accurate sequencing, haplotyping, and de novo assembly. Genome Res., 2019; 29(5): 798–808. doi:10.1101/gr.245126.118. 30940689 PMC6499310

[4] Chen Y SOAPnuke: A MapReduce acceleration-supported software for integrated quality control and preprocessing of high-throughput sequencing data. Gigascience, 2018; 7(1): gix120. doi:10.1093/gigascience/gix120. 29220494 PMC5788068

[5] Chen C-J HiC-Pro: an optimized and flexible pipeline for Hi-C data processing. Genome Biol., 2015; 16: 259. doi:10.1186/s13059-015-0831-x. 26619908 PMC4665391

[6] Vurture GW GenomeScope: Fast reference-free genome profiling from short reads. Bioinformatics, 2017; 33(14): 2202–2204. doi:10.1093/bioinformatics/btx153. 28369201 PMC5870704

[7] BGI-QingDao. stlfr2supernova_pipeline. 2021; https://github.com/BGI-Qingdao/stlfr2supernova_pipeline.

[8] Wong KHY, Levy-Sakin M, Kwok PY, De novo human genome assemblies reveal spectrum of alternative haplotypes in diverse populations. Nat. Commun., 2018; 9: 3040. doi:10.1038/s41467-018-05513-w. 30072691 PMC6072799

[9] Durand NC Juicer provides a one-click system for analyzing loop-resolution Hi-C experiments. Cell Syst., 2016; 3(1): 95–98. doi:10.1016/j.cels.2016.07.002. 27467249 PMC5846465

[10] Dudchenko O De novo assembly of the Aedes aegypti genome using Hi-C yields chromosome-length scaffolds. Science, 2017; 356(6333): 92–95. doi:10.1126/science.aal3327. 28336562 PMC5635820

[11] Waterhouse RM, Seppey M, Sim FA, Ioannidis P, BUSCO applications from quality assessments to gene prediction and phylogenomics. Letter Fast Track, 2017; doi:10.1093/molbev/msx319. PMC5850278 29220515

[12] Wang Y MCScanX: a toolkit for detection and evolutionary analysis of gene synteny and collinearity. Nucleic Acids Res., 2012; 40(7): e49. doi:10.1093/nar/gkr1293. 22217600 PMC3326336

[13] Meng G, Li Y, Yang C, Liu S, MitoZ: A toolkit for animal mitochondrial genome assembly, annotation and visualization. Nucleic Acids Res., 2019; 47(11): e63. doi:10.1093/nar/gkz173. 30864657 PMC6582343

[14] Tillich M GeSeq – versatile and accurate annotation of organelle genomes. Nucleic Acids Res., 2017; 45(W1): W6–W11. doi:10.1093/nar/gkx391. 28486635 PMC5570176

[15] Tarailo-Graovac M, Chen N, Using RepeatMasker to identify repetitive elements in genomic sequences. Curr. Protoc. Bioinformatics, 2009; doi:10.1002/0471250953.bi0410s25. 19274634

[16] Carrillo-Avila M, Resende EK, Marques DKS, Galetti PM, Tandem repeats finder: a program to analyze DNA sequences. Conserv. Genet., 2009; 25: 4.10.1–4.10.14. doi:10.1590/S1679-62252007000200018.

[17] Bao W, Kojima KK, Kohany O, Repbase Update, a database of repetitive elements in eukaryotic genomes. Mob. DNA, 2015; 6: 11. doi:10.1186/s13100-015-0041-9. 26045719 PMC4455052

[18] Xu Z, Wang H, LTR_FINDER: an efficient tool for the prediction of full-length LTR retrotransposons. Nucleic Acids Res., 2007; 35(2): W265–W268. doi:10.1093/nar/gkm286. 17485477 PMC1933203

[19] Storer J, Hubley R, Rosen J, Wheeler TJ, Smit AF, The Dfam community resource of transposable element families, sequence models, and genome annotations. Mob. DNA, 2021; 12: 2. doi:10.1186/s13100-020-00230-y. 33436076 PMC7805219

[20] Stanke M, Schöffmann O, Morgenstern B, Waack S, Gene prediction in eukaryotes with a generalized hidden Markov model that uses hints from external sources. BMC Bioinform., 2006; 7: 62. doi:10.1186/1471-2105-7-62. PMC1409804 16469098

[21] Stanke M, Keller O, Gunduz I, Hayes A, Waack S, Morgenstern B, AUGUSTUS: ab initio prediction of alternative transcripts. Nucleic Acids Res., 2006; 34(2): W435–W439. doi:10.1093/nar/gkl200. 16845043 PMC1538822

[22] Doerks T, Copley RR, Schultz J, Ponting CP, Bork P, Systematic identification of novel protein domain families associated with nuclear functions. Genome Res., 2002; 12(1): 47–56. doi:10.1101/gr.203201. 11779830 PMC155265

[23] Lewis S Creating a honey bee consensus gene set. Genome Biol., 2002; 3: research0082.1. doi:10.1186/gb-2002-3-12-research0082. 17241472 PMC1839126

[24] Bairoch A, The SWISS-PROT protein sequence database and its supplement TrEMBL in 2000. Nucleic Acids Res., 2000; 28(1): 45–48. doi:10.1093/nar/28.1.45. 10592178 PMC102476

[25] Habermann BH, Villaveces JM, Jimenez RC, KEGGViewer, a BioJS component to visualize KEGG pathways. F1000Research, 2014; 3: 43, doi:10.12688/f1000research.3-43.v1. 24715980 PMC3954160

[26] Jones P InterProScan 5: Genome-scale protein function classification. Bioinformatics, 2014; 30(9): 1236–1240. doi:10.1093/bioinformatics/btu031. 24451626 PMC3998142

[27] Altschul SF, Gish W, Miller W, Myers EW, Lipman DJ, Basic local alignment search tool. J. Mol. Biol., 1990; 215(3): 403–410. doi:10.1016/S0022-2836(0580360-2. 2231712

[28] Ruan J TreeFam: 2008 update. Nucleic Acids Res., 2007; 36(suppl 1): D735–D740. doi:10.1093/nar/gkm1005. 18056084 PMC2238856

[29] Guindon S, Dufayard JF, Lefort V, Anisimova M, Hordijk W, Gascuel O, New algorithms and methods to estimate maximum-likelihood phylogenies: Assessing the performance of PhyML 3.0. Syst. Biol., 2010; 59(3): 307–321. doi:10.1093/sysbio/syq010. 20525638

[30] Kimura T Leucophores are similar to xanthophores in their specification and differentiation processes in medaka. Proc. Natl Acad. Sci. USA, 2014; 111(20): 7343–7348. doi:10.1073/pnas.1311254111. 24803434 PMC4034200

[31] Li C Genome data of the bicolor angelfish (*Centropyge bicolor*). GigaScience Database. 2020; 10.5524/100802.

